# Predictive immunohistochemical features for tumour response to chemoradiotherapy in rectal cancer

**DOI:** 10.1002/bjs5.50251

**Published:** 2020-02-05

**Authors:** E. Shinto, J. Omata, A. Sikina, A. Sekizawa, Y. Kajiwara, K. Hayashi, Y. Hashiguchi, K. Hase, H. Ueno

**Affiliations:** ^1^ Department of Surgery National Defense Medical College Tokorozawa Japan; ^2^ Department of Radiology National Defense Medical College Tokorozawa Japan; ^3^ Department of Surgery Self‐Defense Forces Central Hospital Tokyo Japan; ^4^ Department of Surgery Teikyo University School of Medicine Tokyo Japan

## Abstract

**Background:**

Reduced expression of cluster of differentiation (CD) 133 and cyclo‐oxygenase (COX) 2, and increased density of CD8+ tumour‐infiltrating lymphocytes, are associated with a favourable tumour response to preoperative chemoradiotherapy (CRT). This study aimed to evaluate these markers in relation to tumour response after preoperative CRT in two rectal cancer cohorts.

**Methods:**

Patients with low rectal cancer who underwent radical resection and preoperative short‐term CRT in 2001–2007 (retrospective cohort) and long‐term CRT in 2011–2017 (prospective cohort) were analysed. Pretreatment biopsies were stained immunohistochemically using antibodies to determine CD133 and COX‐2 expression, and increased CD8+ density. Outcome measures were tumour regression grade (TRG), tumour downstaging and survival.

**Results:**

For 95 patients in the retrospective cohort, the incidence of TRG 3–4 was 67 per cent when two or three immunohistochemistry (IHC) features were present, but only 20 per cent when there were fewer features (*P* < 0·001). The incidence of tumour downstaging was higher in patients with at least two IHC features (43 *versus* 22 per cent with fewer features; *P* = 0·029). The 49 patients in the prospective cohort had similar rates to those in the retrospective cohort (TRG 3–4: 76 per cent for two or more IHC features *versus* 25 per cent with fewer features, *P* < 0·001; tumour downstaging: 57 *versus* 25 per cent respectively, *P* = 0·022). Local recurrence‐free survival rates in patients with more or fewer IHC features were similar in the retrospective and prospective cohort (*P* = 0·058 and *P* = 0·387 respectively).

**Conclusion:**

Assessment of CD133, COX‐2 and CD8 could be useful in predicting a good response to preoperative CRT in patients with lower rectal cancer undergoing neoadjuvant therapy. Further studies are needed to validate the results in larger cohorts and investigate a survival benefit.

## Graphical abstract

The combination of reduced expression of cluster of differentiation (CD) 133 and cyclo‐oxygenase (COX) 2 and increased density of CD8+ tumour‐infiltrating lymphocytes is a significant predictor of tumour response to preoperative chemoradiotherapy (CRT). This finding may be clinically useful for determining chemoradiosensitive cancers.

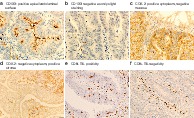

Immunoistochemistry features of tumor regression in rectal cancer

## Abstract (es)

**Antecedentes:**

La expresión reducida de CD133 and COX‐2, y un aumento en la densidad de los linfocitos infiltrantes del tumor CD8+ se han asociado recientemente con una respuesta favorable del tumor a la quimiorradioterapia preoperatoria (*preoperative chemoradiotherapy*, CRT). Este estudio evaluó estos marcadores respecto a la respuesta del tumor tras CRT preoperatoria en dos cohortes de cáncer colorrectal.

**Métodos:**

Se analizaron pacientes con cáncer de recto bajo sometidos a resección radical y CRT preoperatoria de corta duración entre 2001–2007 (cohorte retrospectiva) y CRT de larga duración entre 2011–2017 (cohorte prospectiva). Se realizó tinción inmunohistoquímica (*immunohistochemical*, IHC) con anticuerpos para CD133, COX‐2 y CD8 en las biopsias previas al tratamiento. Las características de interés incluyeron la disminución en las expresiones de CD133 y COX‐2, y la densidad aumentada de CD8+. Las variables de interés fueron los grados de regresión tumoral (*tumour regression grades*, TRG) de acuerdo con Rödel, la reducción del estadio tumoral y las supervivencias.

**Resultados:**

La cohorte retrospectiva incluyó 95 pacientes. En este subgrupo, la incidencia de TRGs 3‐4 fue del 66,7% en pacientes con dos o tres características de la IHC, mientras que solo fue del 20,0% en pacientes con ninguna o con una característica (*P* < 0,001). Además, la incidencia de disminución del estadio tumoral fue más alta en pacientes que mostraban al menos dos características IHC (43,3%) que en los controles (21,5%; *P* = 0,029). En la cohorte prospectiva se incluyeron 49 pacientes y la incidencia de estos hallazgos fue similar (TRG 3‐4, 76,2% en ≥ 2 características IHC *versus* 25,0% en los controles, *P* < 0,001; disminución del estadio tumoral, 57,1% en ≥ 2 características IHC *versus* 25,0% en los controles, *P* = 0,022). La supervivencia libre de recidiva local fue similar en las cohortes retrospectiva y prospectiva, cuando se compararon subgrupos de acuerdo con las características IHC (*P* = 0,058 y 0,387, respectivamente)

**Conclusión:**

Este estudio sugiere que la evaluación de CD133, COX‐2 y CD8 podría ser útil para la predicción de una buena respuesta a la CRT preoperatoria en pacientes con cáncer de recto bajo sometidos a tratamiento neoadyuvante. Se necesitan estudios adicionales para validar los resultados en amplias cohortes e investigar el beneficio en la supervivencia.

## Introduction

Preoperative chemoradiotherapy (CRT) is currently the standard for locally advanced rectal cancer, and aims to lead to tumour regression, downstaging^1^, ^2^, ^3^ and increased resectability^1^, ^2^, ^3^, ^4^. However, neoadjuvant CRT has been associated with postoperative complications including anastomotic leakage and worse anal sphincter function following surgery^5^, ^6^, ^7^. Thus, the investigation of features associated with patient responsiveness is essential to avoid unnecessary treatment.

Reduced expression of cluster of differentiation (CD) 133 and cyclo‐oxygenase (COX) 2, and increased density of CD8+ intraepithelial tumour‐infiltrating lymphocytes (TILs) in biopsy specimens obtained from colonoscopy before preoperative CRT, have been reported to be predictive markers of good tumour response^8^, ^9^. CD133 has been considered a marker of cancer stem cells associated with several tumours, including in colorectal cancer^10^, ^11^, and increasing evidence^12^, ^13^ has demonstrated that these cells are associated with resistance to chemotherapy and radiotherapy. However, COX‐2 promotes the radioresistance of cancer cells via p38/mitogen‐activated protein kinase‐mediated cellular antiapoptosis^14^, and selective COX‐2 inhibitors reportedly increase the susceptibility of tumours to radiation by inhibiting DNA repair processes^15^. In addition, COX‐2 is a powerful angiogenesis‐inducible factor^16^, and induces radioresistance in cancer cells efficiently by increasing blood supply. Further, CD8+ TILs have been reported to affect prognosis positively^17^, possibly indicating that the density of CD8+ TILs is a crucial parameter for determining immunocompetence. Some studies^18^, ^19^ have also demonstrated that radiotherapy and chemotherapy are more efficient in immunocompetent conditions.

With this background, the research hypothesis investigated in the present study was that increased density of CD8+ TILs and reduced expression of CD133 and COX‐2 may predict tumour response to preoperative CRT. The study aimed to assess these features in a retrospective cohort treated with short‐term CRT and a prospective cohort that received long‐term CRT.

## Methods

### Retrospective cohort

The study was approved by the internal review board at the National Defense Medical College; all patients consented to the collection and study of specimens.

Details of all consecutive patients with stage II–IV rectal cancer undergoing preoperative CRT followed by total mesorectal excision between September 2001 and October 2007 at the National Defense Medical College Hospital, a general hospital affiliated to the medical college in Japan, were reviewed and included.

Preoperative CRT was indicated when the distal margin of the tumour was located below the peritoneal reflection, with a preoperative diagnosis of cT3–4 status, obtained using digital examination, colonoscopy, barium enema and MRI. CT was used to determine the extent of extrapelvic tumour spread. A short‐axis value of 5 mm was used as the cut‐off point for determining lymph node metastasis: 5 mm or more and less than 5 mm were regarded as metastasis‐positive and metastasis‐negative respectively. Tumour size was estimated from lateral X‐ray images taken during barium enema in the pretreatment stage. During this period, patients were treated using short‐term preoperative CRT (20 Gy (5 daily doses of 4 Gy) and tegafur–uracil 400 mg/day for 7 days throughout the period of irradiation), followed by total mesorectal excision. In all patients, two opposing fields were used to treat the entire treatment area, which included the anal canal, primary tumour, mesorectal and presacral lymph nodes, and lymph nodes along the internal iliac vessels, those up to the upper border of the fifth lumbar vertebra, and those at the obturator foramina. Data were obtained from medical records and analysed retrospectively.

### Prospective cohort

The study was registered at the University Hospital Medical Education Network clinical trial registry (study ID: UMIN000011993 and UMIN000013486) and received approval from the internal review board. Signed informed consent was obtained from all patients before enrolment. All consecutive patients with stage II–III rectal cancer undergoing long‐term preoperative CRT between July 2011 and April 2017, followed by surgery, were included. The neoadjuvant approach was changed because of a temporary increase in reports of adverse events associated with short‐term preoperative CRT regimens^5^, ^20^, ^21^. Patients were treated with long‐term preoperative CRT (45 Gy (25 daily doses of 1·8 Gy), and S‐1 and irinotecan), followed by total mesorectal excision. Preoperative CRT was indicated for patients with cT3–4 rectal cancer where the distal margin was located below the peritoneal reflection.

Pretreatment assessment procedures were similar to those used in the retrospective study; however, in the prospective cohort patients with stage IV tumours were excluded. S‐1 was administered orally on days 1–5, 8–12, 22–26 and 29–33, based on body surface area (BSA): BSA below 1·25 m^2^, 80 mg/day; BSA 1·25 to less than 1·5 m^2^, 100 mg/day; and BSA 1·5 m^2^ or above, 120 mg/day. Irinotecan was administered as a continuous intravenous infusion on days 1, 8, 22 and 29^22^. A four‐field box technique was used, and the treatment field of radiotherapy was as described previously^23^. The superior margin of the typical irradiation field was set at the level between the fifth lumbar and first sacral vertebra. The inferior margin was set at 3–4 cm below the inferior edge of the primary lesion, as defined principally by a line to the inferior margin of the ischial tuberosity. Lateral margins were 1 cm lateral to the lesser pelvis cavity. The anterior margin was defined as the posterior margin of the pubic symphysis, and the posterior margin was defined as the centre of the sacral bone, as observed from the lateral view. Patient data were collected prospectively, and survival analyses were performed in 2019.

### Immunohistochemistry

**Figure 1:**
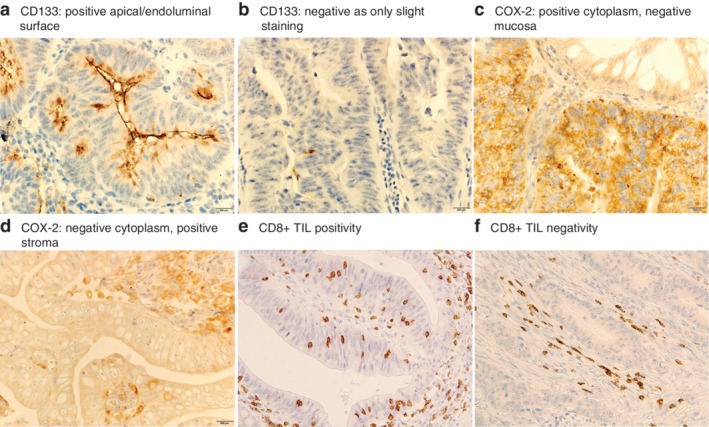
Microscopic features of rectal cancer in biopsy specimens stained for CD133, COX‐2 and CD8 **a** The apical/endoluminal surface of cancer cells is positive for cluster of differentiation (CD) 133. **b** Slight staining of CD133, but categorized as negative. **c** The entire cytoplasm of cancer cells is positive for cyclo‐oxygenase (COX) 2, but the adjacent normal mucosa is negative. **d** The cytoplasm of cancer cells is almost negative for COX‐2, but surrounding stromal cells are positive. **e** CD8+ tumour‐infiltrating lymphocytes (TILs) are prevalent in the intraepithelial compartment, indicating CD8+ TIL positivity. **f** CD8+ lymphocytes are observed mainly in the stroma, which was assumed as CD8+ TIL negativity (magnification for all panels 400 ×).

Pretreatment biopsy specimens obtained using colonoscopy were evaluated using immunostaining for CD133 (clone AC133, dilution 1 : 30; Miltenyi Biotec, Gladbach, Germany), COX‐2 (clone CX229, dilution 1 : 100; Cayman Chemical, Ann Arbor, Michigan, USA) and CD8 (clone C8/144B, dilution 1 : 50; DakoCytomation, Glostrup, Denmark), according to a previously reported procedure^8^. Apical/endoluminal surface staining for CD133 and cytoplasmic staining for COX‐2 in cancer cells were regarded as positive immunoreactivity. CD133 grade was evaluated using the percentage of immuno‐positive cancer cells from the total cancer cells in biopsy specimens. If at least 20 per cent of cancer cells exhibited a positive apical/endoluminal surface staining, they were considered as immunopositive for CD133 (*Fig*. 1 *a,b*)^24^. Immunopositivity for COX‐2 was scored semiquantitatively, and staining intensity and distribution were assessed as follows: staining intensity was scored as 0 (negative), 1 (weak), 2 (medium) and 3 (strong), whereas the scoring for staining distribution was 0 (0 per cent), 1 (1–25 per cent), 2 (26–50 per cent), 3 (51–75 per cent) and 4 (76–100 per cent). Tumours with sum scores (0–7) of 3 or more were considered positive (*Fig*. 1 *c,d*)^8^.

For CD8, cytoplasmic staining was regarded as positive immunoreactivity; however, during evaluation, non‐nucleated small fragments were not enumerated. For CD8+ TILs, positively stained cells that were present entirely in epithelial compartments were counted in the densest field observed at 40 × magnification, and fields with counts of six or more were designated as positive (*Fig*. 1 *e,f*)^9^.

### Outcome measures

Outcome measures were tumour regression grade (TRG), tumour downstaging and survival. TRG was assessed semiquantitatively using haematoxylin and eosin‐stained slides, as described previously^25^. Briefly, TRG was categorized as follows: TRG 0, no regression; TRG 1, dominant tumour mass with obvious fibrosis in 25 per cent or less of the tumour; TRG 2, dominant tumour mass with obvious fibrosis in 26–50 per cent of the tumour; TRG 3, dominant fibrosis outgrowing the tumour (more than 50 per cent); and TRG 4, only a fibrotic mass with no viable cancer cells. Tumour downstaging was defined as pathological findings of ypT0–2, because all cancers had been estimated to be cT3–4 according to inclusion criteria. Local recurrence‐free survival was defined as the time from surgery to local recurrence in patients with rectal cancer, whereas relapse‐free survival was defined as the time to the first relapse or death from any cause^26^.

All patients received regular follow‐up at the outpatient clinic. Physical examination and blood testing, including testing for carcinoembryonic antigen and carbohydrate antigen 19‐9 levels, were performed every 3 months. Contrast CT was done every 6 months. If patients did not attend the clinic, follow‐up was by telephone interviews once a year.

### Statistical analysis

Categorical variables were compared using the χ^2^ or Fisher's exact test, whereas unpaired *t* tests were used to compare normally distributed continuous variables. The association between pretreatment parameters (immunohistochemistry (IHC) and clinical/pathological variables) and TRG was tested using logistic regression, with a forced entry procedure to determine the hazard ratio (HR) and 95 per cent confidence interval. Cut‐off values for clinical/pathological variables (age, tumour size and distance to anal verge) were based on receiver operating characteristic (ROC) curve analysis of the probability of TRG 3 or 4. Survival probabilities were calculated using the Kaplan–Meier method, with comparisons made using the log rank test.

All statistical analyses were performed using the JMP® 12 software (SAS Institute, Cary, North Carolina, USA). *P* < 0·050 was considered statistically significant.

## Results

All clinical/pathological and IHC features for the two cohorts are shown in *Table* 1.

**Table 1:** Clinicopathological patient features and their association with immunoreactivity

	Retrospective cohort				Prospective cohort			
	Total (*n* = 95)	CD133 low (*n* = 74)	COX‐2 low (*n* = 20)	CD8+ TIL high (*n* = 23)	Total (*n* = 49)	CD133 low (*n* = 31)	COX‐2 low (*n* = 15)	CD8+ TIL high (*n* = 18)
**Age (years)** *	61·5(8·7)	61·4(9·1)	63·8(9·7)	63·8(7·3)	62·1(10·5)	63·4(9·0)	60·5(11·3)	62·6(7·4)
**Sex**								
M	66 (69)	53 (72)	12 (60)	14 (61)	34 (69)	22 (71)	10 (67)	10 (56)
F	29 (31)	21 (28)	8 (40)	9 (39)	15 (31)	9 (29)	5 (33)	8 (44)
**Pretreatment cT category**								
cT3	91 (96)	70 (95)	18 (90)	23 (100)	47 (96)	31 (100)	15 (100)	18 (100)
cT4	4 (4)	4 (5)	2 (10)	0 (0)	2 (4)	0 (0)	0 (0)	0 (0)
**Pretreatment cN category**								
cN0	39 (41)	32 (43)	11 (55)	12 (52)	13 (27)	8 (26)	4 (27)	4 (22)
cN1–2	56 (59)	42 (57)	9 (45)	11 (48)	36 (73)	23 (74)	11 (73)	14 (78)
**Pretreatment tumour size (mm)** *	45·4(14·5)	44·2(14·0)	45·9(17·3)	42·3(12·8)	43·1(13·9)	41·8(15·0)	40·3(12·5)	41·7(8·7)
**Distance to anal verge (mm)** *	44·5(18·3)	42·3(19·1)	42·3(18·4)	45·0(19·8)	44·5(24·2)	41·6(26·0)	42·7(27·1)	52·2(21·0)
**Tumour differentiation in pretreatment biopsy specimen**								
Well/moderate†	89 (94)	68 (92)	18 (90)	20 (87)	43 (88)	25 (81)	13 (87)	17 (94)
Poor, mucinous or signet‡	6 (6)	6 (8)	2 (10)	3 (13)	6 (12)	6 (19)	2 (13)	1 (6)
**Pretreatment CEA level (ng/ml)**								
≤ 5·3§	69 (73)	55 (74)	16 (80)	18 (78)	31 (63)	20 (65)	9 (60)	15 (83)
> 5·3	26 (27)	19 (26)	4 (20)	5 (22)	18 (37)	11 (35)	6 (40)	3 (17)
**Pretreatment CA19‐9 level (units/ml)**								
≤ 37§	82 (86)	64 (86)	18 (90)	17 (74)	40 (82)	24 (77)	12 (80)	15 (83)
> 37	13 (14)	10 (14)	2 (10)	6 (26)	9 (18)	7 (23)	3 (20)	3 (17)
**Time from CRT to resection (days)** *	31·8(8·6)	31·8(9·2)	30·4(6·2)	29·0(5·6)	49·6(8·0)	49·8(9·0)	46·8(8·2)	49·4(7·5)
**Tumour differentiation in resected specimen** *								
pCR	3 (3)	3 (4)	3 (15)	3 (13)	8 (16)	6 (19)	4 (27)	5 (28)
Well/moderate†	85 (89)	64 (86)	15 (75)	18 (78)	36 (73)	20 (65)	10 (67)	12 (67)
Poor, mucinous or signet‡	7 (7)	7 (9)	2 (10)	2 (9)	5 (10)	5 (16)	1 (7)	1 (6)
**pT category**								
pCR	3 (3)	3 (4)	3 (15)	3 (13)	8 (16)	6 (19)	4 (27)	5 (28)
pTis and pT1–2	24 (25)	20 (27)	4 (20)	9 (39)	11 (22)	8 (26)	4 (27)	5 (28)
pT3–4	68 (72)	51 (69)	13 (65)	11 (48)	30 (61)	17 (55)	7 (47)	8 (44)
**pN category**								
pN0	46 (48)	37 (50)	13 (65)	14 (61)	32 (65)	21 (68)	9 (60)	13 (72)
pN1–2	49 (52)	37 (50)	7 (35)	9 (39)	17 (35)	10 (32)	6 (40)	5 (28)
**TRG**								
0	1 (1)	0 (0)	0 (0)	0 (0)	0 (0)	0 (0)	0 (0)	0 (0)
1	24 (25)	15 (20)	0 (0)	2 (9)	12 (24)	10 (32)	4 (27)	4 (22)
2	37 (39)	27 (36)	6 (30)	7 (30)	14 (29)	6 (19)	0 (0)	1 (6)
3	30 (32)	29 (39)	11 (55)	11 (48)	15 (31)	9 (29)	7 (47)	8 (44)
4 (pCR)	3 (3)	3 (4)	3 (15)	3 (13)	8 (16)	6 (19)	4 (27)	5 (28)

Values in parentheses are percentages unless indicated otherwise;

* values are mean(s.d.).

† Well or moderately differentiated tubular adenocarcinoma;

‡ poorly differentiated or mucinous adenocarcinoma, or signet ring cell carcinoma.

§ Within normal limit. CD, cluster of differentiation; COX, cyclo‐oxygenase; TIL, tumour‐infiltrating lymphocyte; CEA, carcinoembryonic antigen; CA, carbohydrate antigen; CRT, preoperative chemoradiotherapy; pCR, pathological complete response; TRG, tumour regression grade.

### Retrospective cohort

Of 101 patients reviewed, six were excluded owing to an insufficient volume of archival paraffin‐embedded tissue blocks from pretreatment biopsy specimens for IHC staining, leaving for data analysis 95 consecutive patients.

The majority of these patients were assessed as having clinical stage II or III disease; four patients presented with distant metastasis, although tumours were determined as resectable. The mean(s.d.) interval between preoperative CRT and surgery was 31·8(8·6) days.

*Table* 2 gives the results of univariable and multivariable analysis for the selection of predictive parameters for a good TRG (3 or 4) after preoperative CRT. Based on multivariable analysis, low expression of CD133 (HR 8·52, 95 per cent c.i. 1·38 to 168·18; *P* = 0·018), low expression of COX‐2 (HR 5·83, 1·68 to 23·39; *P* = 0·005) and increased CD8+ TIL density (HR 3·01, 0·93 to 10·15; *P* = 0·066) were independent or marginally independent parameters that influenced TRG. These data suggest that CD133, COX‐2 and CD8+ TILs are eligible as constituents of the predictive model.

**Table 2:** Univariable and multivariable analysis of clinicopathological parameters for tumour regression grade 3–4 in the retrospective cohort

	Univariable analysis		Multivariable analysis‡	
	Odds ratio	*P*	Odds ratio	*P*
CD133 (low *versus* high)	15·24 (2·93, 280·48)	< 0·001	8·52 (1·38, 168·18)	0·018
COX‐2 (low *versus* high)	6·88 (2·41, 21·86)	< 0·001	5·83 (1·68, 23·39)	0·005
CD8+ TIL (high *versus* low)	4·34 (1·64, 12·04)	0·003	3·01 (0·93, 10·15)	0·066
Age (> 68 *versus* ≤ 68 years)*	1·71 (0·66, 4·39)	0·262		
Sex (M *versus* F)	1·01 (0·41, 2·61)	0·973		
Pretreatment cT category (cT3 *versus* cT4)	1·62 (0·20, 33·41)	0·668		
Pretreatment cN category (cN0 *versus* cN1–2)	2·86 (1·16, 7·19)	0·023	2·16 (0·72, 6·58)	0·167
Pretreatment tumour size (≤ 52·6 *versus* > 52·6 mm)*	4·09 (1·25, 18·56)	0·018	2·37 (0·60, 11·99)	0·226
Distance to anal verge (≤ 40 *versus* > 40 mm)*	3·00 (1·27, 7·35)	0·012	2·23 (0·78, 6·70)	0·135
Tumour differentiation in pretreatment biopsy specimen (well/moderate *versus* poor, mucinous or signet)†	0·24 (0·03, 1·31)	0·100		
Pretreatment CEA level (≤ 5·3 *versus* > 5·3 ng/ml)	1·64 (0·63, 4·68)	0·320		
Pretreatment CA19‐9 level (≤ 37 *versus* > 37 units/ml)	1·23 (0·37, 4·86)	0·744		

Values in parentheses are 95 per cent confidence intervals.

* Cut‐off determined by receiver operating characteristic (ROC) curve analysis.

† Well or moderately differentiated tubular adenocarcinoma *versus* moderately differentiated or mucinous tubular adenocarcinoma, or signet ring cell carcinoma. CD, cluster of differentiation; COX, cyclo‐oxygenase; TIL, tumour‐infiltrating lymphocyte; CEA, carcinoembryonic antigen; CA, carbohydrate antigen.

‡ Logistic multivariable analysis of variables with *P* < 0·050 in univariable analysis.

### Prospective cohort

Forty‐nine patients were enrolled prospectively. Between July 2011 and May 2013, 13 patients received 80 mg/m^2^ irinotecan (UMIN000011993). However, seven of these 13 patients developed grade III or higher adverse events, such as diarrhoea (3 patients), neutropenia (4) and anorexia (1). Thus, from patient 14 onwards, only S‐1 was administered between March 2014 and April 2017 (UMIN000013486). The mean(s.d.) interval from preoperative CRT to surgery was 49·6(8·0) days.

### Tumour regression grade and immunohistochemistry analysis

Statistical analysis revealed that TRG 3–4 was associated with positive IHC findings for the three IHC features, comprising reduced expression of CD133 (*P* = 0·001), reduced expression of COX‐2 (*P* < 0·001) and increased density of CD8+ TILs (*P* = 0·003) in the retrospective cohort. This was partially confirmed in the prospective enrolment, where TRG 3–4 was associated with the reduced expression of COX‐2 (*P* = 0·014) and increased density of CD8+ TILs (*P* = 0·007), but not reduced expression of CD133 (*P* = 0·790) (*Table* 3).

**Table 3:** Predictive values for three markers used in pretreatment biopsy specimens for tumour regression grade and downstaging

	Patients with TRG 3–4	*P* *	Patients with downstaged tumour (≤ ypT2)	*P* *
**Retrospective cohort**				
CD133 (high *versus* low)	1 of 21 (5) *versus* 32 of 74 (43)	0·001	4 of 21 (19) *versus* 23 of 74 (31)	0·281
COX‐2 (high *versus* low)	19 of 75 (25) *versus* 14 of 20 (70)	< 0·001	20 of 75 (27) *versus* 7 of 20 (35)	0·463
CD8+ TIL (high *versus* low)	14 of 23 (61) *versus* 19 of 72 (26)	0·003	12 of 23 (52) *versus* 15 of 72 (21)	0·004
**Prospective cohort**				
CD133 (high *versus* low)	8 of 18 (44) *versus* 15 of 31 (48)	0·790	5 of 18 (28) *versus* 14 of 31 (45)	0·229
COX‐2 (high *versus* low)	12 of 34 (35) *versus* 11 of 15 (73)	0·014	11 of 34 (32) *versus* 8 of 15 (53)	0·165
CD8+ TIL (high *versus* low)	13 of 18 (72) *versus* 10 of 31 (32)	0·007	10 of 18 (56) *versus* 9 of 31 (29)	0·066

Values in parentheses are percentages. TRG, tumour regression grade; CD, cluster of differentiation; COX, cyclo‐oxygenase; TIL, tumour‐infiltrating lymphocyte.

* χ^2^ or Fisher's exact test.

Increased density of CD8+ TILs was also associated with a pathological report consistent with ypT0–2, although with limited value in prospective patients (retrospective cohort, *P* = 0·004; prospective cohort, *P* = 0·066). However, expression levels of CD133 and COX‐2 showed no statistical significance (*Table* 3).

When the number of IHC features of the three markers was assessed (*Table* 4), retrospective data indicated that the incidence of TRG 3–4 was 67 per cent (20 of 30) in patients with two or three features, but only 20 per cent (13 of 65) in patients with no or one feature (*P* < 0·001) (positive predictive value (PPV), 67 per cent; negative predictive value (NPV) 80 per cent). The rate of tumour downstaging was higher in patients with two or three features (13 of 30, 43 per cent) than in those with fewer features (14 of 65, 22 per cent) (*P* = 0·029).

**Table 4:** Predictive values for total number of positive immunohistochemistry markers in pretreatment biopsy specimens for tumour regression grade and downstaging

No. of positive IHC markers	Patients with TRG 3–4		*P* *	Patients with downstaged tumour (≤ ypT2)		*P* *
**Retrospective cohort**						
0	1 of 17 (6)	13 of 65 (20)	< 0·001	3 of 17 (18)	14 of 65 (22)	0·029
1	12 of 48 (25)			11 of 48 (23)		
2	12 of 21 (57)	20 of 30 (67)		8 of 21 (38)	13 of 30 (43)	
3	8 of 9 (89)			5 of 9 (56)		
**Prospective cohort**						
0	3 of 11 (27)	7 of 28 (25)	< 0·001	1 of 11 (9)	7 of 28 (25)	0·022
1	4 of 17 (24)			6 of 17 (35)		
2	13 of 16 (81)	16 of 21 (76)		10 of 16 (63)	12 of 21 (57)	
3	3 of 5 (60)			2 of 5 (40)		

Values in parentheses are percentages. IHC, immunohistochemical; TRG, tumour regression grade.

* Comparison between patients with two or three IHC markers and those with no or one factor (χ^2^ or Fisher's exact test).

Similarly, prospective data demonstrated that the incidence of TRG 3–4 was 76 per cent (16 of 21) in patients with two or three markers, but only 25 per cent (7 of 28) in those with no or one factor (*P* < 0·001) (PPV, 76 per cent; NPV, 75 per cent). The rate of tumour downstaging was also higher in patients with more features (12 of 21, 57 per cent) than in those with fewer (7 of 28, 25 per cent) (*P* = 0·022) (*Table* 4).

Univariable analysis of data from the prospective cohort indicated that two or three IHC markers (*P* < 0·001) and pretreatment tumour size of 46·0 mm or less (*P* = 0·015) were significantly associated with a good tumour response (TRG 3–4), whereas other pretreatment parameters failed to show significance. In multivariable analysis of these two variables, both the IHC factor (HR 9·13, 95 per cent c.i. 2·44 to 41·00; *P* < 0·001) and pretreatment tumour size (HR 4·38, 1·04 to 22·50; *P* = 0·044) were independently associated with a good tumour response.

### Survival analysis

Of 87 patients in the retrospective cohort who had an R0 resection, 26 died a median of 47·0 (range 2·1–149·5) months after surgery. The median duration of follow‐up for the remaining 61 patients was 78·2 (29·0–191·8) months. The difference between 5‐year local recurrence‐free survival rate in patients with two or three features (100 per cent) and those with no or one feature (88 per cent) was of borderline significance (*P* = 0·058). In addition, the 5‐year relapse‐free survival rate did not differ in these two subgroups (80 *versus* 68 per cent respectively; *P* = 0·501).

Among the 44 patients with an R0 resection in the prospective cohort, four died a median of 48·2 (range 12·3–66·3) months after surgery. The median duration of follow‐up for the remaining 40 patients was 38·8 (21·3–87·4) months. Neither the 5‐year local recurrence‐free survival rate (100 per cent for patients with 2 or 3 features *versus* 96 per cent for those with 0 or 1 feature; *P* = 0·387) nor the relapse‐free survival rate (100 *versus* 76 per cent respectively; *P* = 0·085) was significantly different.

## Discussion

In recent years, several studies have evaluated the potential of molecular biomarkers to predict tumour response to CRT. A review of the literature, including several studies reporting on gene expression profiles associated with tumour response to CRT, concluded that there was little consistency with respect to the selected genes for determining CRT sensitivity^27^. This variation could be ascribed to the use of different methodologies. Some studies^28^, ^29^, ^30^, ^31^, ^32^, ^33^ also examined genetic/epigenetic changes or protein expression levels, although research is still at an early stage. Nevertheless, large‐scale validation studies of predictive markers are necessary before incorporating such methodologies into future clinical practice. Although a number of promising predictive classifiers have been proposed, successful validation has not yet been achieved.

In the present study, findings from the retrospective cohort indicated that expression of CD133 and COX‐2, and the density of CD8+ TILs were significant predictors of tumour response to preoperative CRT. However, the prospective study, although limited by sample size, disclosed excellent predictive value for the three markers, except for CD133 immunoreactivity. However, the interval between preoperative CRT and surgery was longer in the long‐term preoperative CRT cohort, and this may have had a negative influence on the radioresistant property of CD133+ cancer cells.

The present study also showed that the increased density of CD8+ TILs is a prominent predictor of tumour downstaging. A previous study^9^ demonstrated that a high density of CD8+ TILs in pretreatment biopsy specimens correlated strongly with enhanced CD8+ lymphocyte aggregation at the tumour margin after preoperative CRT. This could suggest that CRT‐induced CD8+ lymphocytes may respond to cancer cells located at the invasive front, resulting in tumour downstaging. Conversely, increased CD8+ lymphocyte aggregation at the tumour margin after CRT was rarely observed in cancers with a low density of CD8+ TILs in pretreatment biopsy specimens^9^, which prevented downstaging. Patients with rectal cancer who did not have preoperative CRT also showed a positive association between the density of CD8+ TILs in biopsy specimens and the level of CD8+ lymphocyte aggregation at the tumour margin. However, the level of CD8+ lymphocyte aggregation at the tumour margin in patients who had preoperative CRT was strikingly higher than that in those who had surgery alone^9^. These data suggest that patients with a high density of CD8+ TILs in pretreatment biopsy specimens demonstrate tumour downstaging as a benefit of preoperative CRT, owing to enhanced immunoreactivity after preoperative CRT at the invasive tumour margin.

Overall, the combined analysis of the three markers could be a powerful tool for identifying the chemoradiosensitivity of patients with lower rectal cancer, consistent with the research hypothesis. Of note, a previous report^8^, ^9^ also demonstrated excellent interobserver agreement for evaluating CD133 and COX‐2 immunostaining, and CD8+ TIL density: 85·4 per cent (κ = 0·68), 92·9 per cent (κ = 0·76) and 89·9 per cent (κ = 0·79) respectively.

This study has some limitations. First, the preoperative CRT regimens adopted and the intervals between preoperative CRT and surgery differed between the prospective and retrospective studies. Second, the characteristics of biopsy specimens may not represent those of whole tumours.

A prospective trial involving patients with only two or three positive findings for treatment using preoperative CRT (study ID: UMIN000026306) is currently ongoing, to obtain more robust findings. The results of this trial will be necessary to determine the clinical usefulness of these proposed IHC markers.

## Acknowledgements

This work was supported partly by the Japan Society for the Promotion of Science KAKENHI (25462074 and 18 K08721). The authors thank Y. Hasumi for expert technical assistance, and acknowledge the contributions of T. Kaji and M. Takano regarding treatment procedures.

*Disclosure*: The authors declare no conflict of interest.

## References

[1] Janjan NA , Khoo VS , Abbruzzese J , Pazdur R , Dubrow R , Cleary KR *et al* Tumor downstaging and sphincter preservation with preoperative chemoradiation in locally advanced rectal cancer: the M. D. Anderson Cancer Center experience. Int J Radiat Oncol Biol Phys 1999; 44: 1027–1038. 10421535 10.1016/s0360-3016(99)00099-1

[2] Theodoropoulos G , Wise WE , Padmanabhan A , Kerner BA , Taylor CW , Aguilar PS *et al* T‐level downstaging and complete pathologic response after preoperative chemoradiation for advanced rectal cancer result in decreased recurrence and improved disease‐free survival. Dis Colon Rectum 2002; 45: 895–903. 12130878 10.1007/s10350-004-6325-7

[3] Kaminsky‐Forrett MC , Conroy T , Luporsi E , Peiffert D , Lapeyre M , Boissel P *et al* Prognostic implications of downstaging following preoperative radiation therapy for operable T3–T4 rectal cancer. Int J Radiat Oncol Biol Phys 1998; 42: 935–941. 9869213 10.1016/s0360-3016(98)00345-9

[4] Chan AK , Wong AO , Langevin J , Jenken D , Heine J , Buie D *et al* Preoperative chemotherapy and pelvic radiation for tethered or fixed rectal cancer: a phase II dose escalation study. Int J Radiat Oncol Biol Phys 2000; 48: 843–856. 11020583 10.1016/s0360-3016(00)00692-1

[5] Birgisson H , Påhlman L , Gunnarsson U , Glimelius B ; Swedish Rectal Cancer Trial Group . Adverse effects of preoperative radiation therapy for rectal cancer: long‐term follow‐up of the Swedish Rectal Cancer Trial. J Clin Oncol 2005; 23: 8697–8705. 16314629 10.1200/JCO.2005.02.9017

[6] Stelzmueller I , Zitt M , Aigner F , Kafka‐Ritsch R , Jäger R , De Vries A *et al* Postoperative morbidity following chemoradiation for locally advanced low rectal cancer. J Gastrointest Surg 2009; 13: 657–667. 19082672 10.1007/s11605-008-0760-z

[7] Gervaz P , Rotholtz N , Pisano M , Kaplan E , Secic M , Coucke P *et al* Quantitative short‐term study of anal sphincter function after chemoradiation for rectal cancer. Arch Surg 2001; 136: 192–196. 11177140 10.1001/archsurg.136.2.192

[8] Shinto E , Hashiguchi Y , Ueno H , Kobayashi H , Ishiguro M , Mochizuki H *et al* Pretreatment CD133 and cyclooxygenase‐2 expression as the predictive markers of the pathological effect of chemoradiotherapy in rectal cancer patients. Dis Colon Rectum 2011; 54: 1098–1106. 21825889 10.1097/DCR.0b013e3182218155

[9] Shinto E , Hase K , Hashiguchi Y , Sekizawa A , Ueno H , Shikina A *et al* CD8+ and FOXP3+ tumor‐infiltrating T cells before and after chemoradiotherapy for rectal cancer. Ann Surg Oncol 2014; 21(Suppl 3): S414–S421. 24566864 10.1245/s10434-014-3584-y

[10] Ricci‐Vitiani L , Lombardi DG , Pilozzi E , Biffoni M , Todaro M , Peschle C *et al* Identification and expansion of human colon‐cancer‐initiating cells. Nature 2007; 445: 111–115. 17122771 10.1038/nature05384

[11] O'Brien CA , Pollett A , Gallinger S , Dick JE . A human colon cancer cell capable of initiating tumour growth in immunodeficient mice. Nature 2007; 445: 106–110. 17122772 10.1038/nature05372

[12] Ong CW , Kim LG , Kong HH , Low LY , Iacopetta B , Soong R *et al* CD133 expression predicts for non‐response to chemotherapy in colorectal cancer. Mod Pathol 2010; 23: 450–457. 20081809 10.1038/modpathol.2009.181

[13] Rich JN . Cancer stem cells in radiation resistance. Cancer Res 2007; 67: 8980–8984. 17908997 10.1158/0008-5472.CAN-07-0895

[14] Lin F , Luo J , Gao W , Wu J , Shao Z , Wang Z *et al* COX‐2 promotes breast cancer cell radioresistance via p38/MAPK‐mediated cellular anti‐apoptosis and invasiveness. Tumour Biol 2013; 34: 2817–2826. 23771849 10.1007/s13277-013-0840-x

[15] Raju U , Ariga H , Dittmann K , Nakata E , Ang KK , Milas L . Inhibition of DNA repair as a mechanism of enhanced radioresponse of head and neck carcinoma cells by a selective cyclooxygenase‐2 inhibitor, celecoxib. Int J Radiat Oncol Biol Phys 2005; 63: 520–528. 16168844 10.1016/j.ijrobp.2005.06.007

[16] Cianchi F , Cortesini C , Bechi P , Fantappiè O , Messerini L , Vannacci A *et al* Up‐regulation of cyclooxygenase 2 gene expression correlates with tumor angiogenesis in human colorectal cancer. Gastroenterology 2001; 121: 1339–1347. 11729113 10.1053/gast.2001.29691

[17] Naito Y , Saito K , Shiiba K , Ohuchi A , Saigenji K , Nagura H *et al* CD8+ T cells infiltrated within cancer cell nests as a prognostic factor in human colorectal cancer. Cancer Res 1998; 58: 3491–3494. 9721846

[18] Apetoh L , Ghiringhelli F , Tesniere A , Obeid M , Ortiz C , Criollo A *et al* Toll‐like receptor 4‐dependent contribution of the immune system to anticancer chemotherapy and radiotherapy. Nat Med 2007; 13: 1050–1059. 17704786 10.1038/nm1622

[19] Plotnikov A , Niego B , Ophir R , Korenstein R , Keisari Y . Effective treatment of mouse metastatic prostate cancer by low electric field enhanced chemotherapy. Prostate 2006; 66: 1620–1630. 16941466 10.1002/pros.20435

[20] Peeters KC , van de Velde CJ , Leer JW , Martijn H , Junggeburt JM , Kranenbarg EK *et al* Late side effects of short‐course preoperative radiotherapy combined with total mesorectal excision for rectal cancer: increased bowel dysfunction in irradiated patients – a Dutch Colorectal Cancer Group study. J Clin Oncol 2005; 23: 6199–6206. 16135487 10.1200/JCO.2005.14.779

[21] Birgisson H , Påhlman L , Gunnarsson U , Glimelius B . Late adverse effects of radiation therapy for rectal cancer – a systematic overview. Acta Oncol 2007; 46: 504–516. 17497318 10.1080/02841860701348670

[22] Sato T , Ozawa H , Hatate K , Onosato W , Naito M , Nakamura T *et al* A phase II trial of neoadjuvant preoperative chemoradiotherapy with S‐1 plus irinotecan and radiation in patients with locally advanced rectal cancer: clinical feasibility and response rate. Int J Radiat Oncol Biol Phys 2011; 79: 677–683. 21035953 10.1016/j.ijrobp.2009.11.007

[23] Sato T , Hayakawa K , Tomita N , Noda M , Kamikonya N , Watanabe T *et al* A multicenter phase I study of preoperative chemoradiotherapy with S‐1 and irinotecan for locally advanced lower rectal cancer (SAMRAI‐1). Radiother Oncol 2016; 120: 222–227. 27317556 10.1016/j.radonc.2016.06.002 PMC5013752

[24] Zhou F , Mu YD , Liang J , Liu ZX , Chen HS , Zhang JF . Expression and prognostic value of tumor stem cell markers ALDH1 and CD133 in colorectal carcinoma. Oncol Lett 2014; 7: 507–512. 24396478 10.3892/ol.2013.1723 PMC3881922

[25] Rödel C , Martus P , Papadoupolos T , Füzesi L , Klimpfinger M , Fietkau R *et al* Prognostic significance of tumor regression after preoperative chemoradiotherapy for rectal cancer. J Clin Oncol 2005; 23: 8688–8696. 16246976 10.1200/JCO.2005.02.1329

[26] Japanese Society for Cancer of the Colon and Rectum . Japanese Classification of Colorectal Carcinoma (3rd English edn). Tokyo: Kanehara, 2019.

[27] Akiyoshi T , Kobunai T , Watanabe T . Predicting the response to preoperative radiation or chemoradiation by a microarray analysis of the gene expression profiles in rectal cancer. Surg Today 2012; 42: 713–719. 22706722 10.1007/s00595-012-0223-8

[28] Erben P , Ströbel P , Horisberger K , Popa J , Bohn B , Hanfstein B *et al* *KRAS* and *BRAF* mutations and PTEN expression do not predict efficacy of cetuximab‐based chemoradiotherapy in locally advanced rectal cancer. Int J Radiat Oncol Biol Phys 2011; 81: 1032–1038. 20947270 10.1016/j.ijrobp.2010.06.043

[29] Garcia‐Aguilar J , Chen Z , Smith DD , Li W , Madoff RD , Cataldo P *et al* Identification of a biomarker profile associated with resistance to neoadjuvant chemoradiation therapy in rectal cancer. Ann Surg 2011; 254: 486–492. 21865946 10.1097/SLA.0b013e31822b8cfa PMC3202983

[30] Russo AL , Ryan DP , Borger DR , Wo JY , Szymonifka J , Liang WY *et al* Mutational and clinical predictors of pathologic complete response in the treatment of locally advanced rectal cancer. J Gastrointest Cancer 2014; 45: 34–39. 24006244 10.1007/s12029-013-9546-y PMC4361942

[31] Tsang JS , Vencken S , Sharaf O , Leen E , Kay EW , McNamara DA *et al* Global DNA methylation is altered by neoadjuvant chemoradiotherapy in rectal cancer and may predict response to treatment – a pilot study. Eur J Surg Oncol 2014; 40: 1459–1466. 25108814 10.1016/j.ejso.2014.06.008

[32] Kikuchi M , Mikami T , Sato T , Tokuyama W , Araki K , Watanabe M *et al* High Ki67, Bax, and thymidylate synthase expression well correlates with response to chemoradiation therapy in locally advanced rectal cancers: proposal of a logistic model for prediction. Br J Cancer 2009; 101: 116–123. 19491899 10.1038/sj.bjc.6605105 PMC2713712

[33] Yokoi K , Yamashita K , Ishii S , Tanaka T , Nishizawa N , Tsutsui A *et al* Comprehensive molecular exploration identified promoter DNA methylation of the *CRBP1* gene as a determinant of radiation sensitivity in rectal cancer. Br J Cancer 2017; 116: 1046–1056. 28291773 10.1038/bjc.2017.65 PMC5396119

